# Olfactory Testing and Gray Matter Volume: A Combined Approach to Predict the Conversion to Alzheimer

**DOI:** 10.3390/brainsci15030310

**Published:** 2025-03-15

**Authors:** Claudia Casadio, Daniela Ballotta, Francesco Ricci, Vanessa Zanelli, Omar Carpentiero, Maria Giulia Corni, Elisa Bardi, Nicola Filippini, Fausta Lui, Paolo Frigio Nichelli, Maria Angela Molinari, Francesca Benuzzi

**Affiliations:** 1 Department of Biomedical, Metabolic and Neural Sciences, University of Modena and Reggio Emilia, 41125 Modena, Italy; claudia.casadio@unimore.it (C.C.); daniela.ballotta@unimore.it (D.B.); francesco.ricci@unimore.it (F.R.); vanessa.zanelli@unimore.it (V.Z.); 269868@studenti.unimore.it (O.C.); fausta.lui@unimore.it (F.L.); paolofrigionichelli@gmail.com (P.F.N.); 2 Department of Integration, AUSL Modena, 41121 Modena, Italy; mariagiuliacorni@gmail.com (M.G.C.); elisabardi7@gmail.com (E.B.); 3 IRCCS San Camillo Hospital, 30126 Venice, Italy; nicola.filippini@hsancamillo.it; 4 AOU Modena, 41126 Modena, Italy; m.molinari@ausl.mo.it

**Keywords:** Mild Cognitive Impairment, Alzheimer’s isease, olfaction, memory

## Abstract

**Background/Objectives**: Olfactory decline is common in normal aging and frequent in neurodegenerative diseases such as Alzheimer’s disease (AD). Therefore, it has been suggested as a marker for the Mild Cognitive Impairment (MCI) progression to AD. Although suggested, the relationship between olfactory deficits and cerebral atrophy in MCI conversion to AD is still debated. This study aims at investigating the olfaction-related morphological and behavioural alterations in MCI in order to understand whether they can predict the progression to AD. **Methods**: Twenty-seven MCI patients and thirty-five healthy controls (HCs) took part in the study, with follow-up showing conversion to AD in thirteen patients (converter-MCI, cMCI). The Burgarth Sniffin’ Sticks Tests (threshold—TT, discrimination—DT, identification—IT) assessed the olfactory capacities. The Voxel-Based Morphometry (VBM) analysis investigated the atrophic patterns. **Results**: The Receiving Operating Characteristics analyses demonstrated that DT and IT could distinguish HC from MCI (DT Area Under Curve—AUC = 0.8; IT AUC = 0.8), as well as cMCI from sMCI (stable) patients (DT AUC = 0.7; IT AUC = 0.6), similarly to memory and executive functions tests. Olfactory performance positively correlated with memory tests in sMCI (all *rho*s ≥ 0.8, all *p*s < 0.01), whereas it positively correlated with executive functions in cMCI (all *rho*s ≥ 0.6, all *p*s < 0.05). VBM results revealed distinct atrophic patterns in cMCI, especially in the olfactory cortex, that were already present at the MCI diagnosis, before AD conversion. A larger volume of the olfactory cortex was associated with better memory and executive functions. **Conclusions**: Quantitative olfactory and morphological patterns represent non-invasive, predictive biomarkers of the MCI progression to AD; thus, their assessments at MCI onset allows earlier interventions for MCI patients.

## 1. Introduction

The interplay between olfaction and memory has been widely considered in various fields, from literature to neurophysiology. Marcel Proust famously narrated the evocative effects of odors in eliciting vivid autobiographical memories through the depiction of the madeleine episode, and the investigation of the neurobiological underpinnings of the olfaction–memory relationship has flourished since [1].

The olfactory system exhibits a unique relationship with memory, primarily due to its shared neuroanatomical substrate within the limbic system. Unlike other sensory modalities, olfactory signals bypass the thalamus and project directly to the primary olfactory cortex, including the piriform cortex, before extending to higher-order regions such as the orbitofrontal cortex and hippocampus. These pathways include structures essential for memory and emotion, notably the amygdala and anterior hippocampus [2], as well as the entorhinal cortex [3,4] and the piriform cortex, which represents a critical node that enables olfactory information processing and its association with past experiences [5]. This finding supports the idea that olfactory stimuli can evoke vivid, emotionally charged memories more effectively than other sensory inputs.

Olfactory functionality naturally declines with age. Indeed, previous studies consistently report a reduction in odor identification, discrimination, and detection thresholds among healthy older adults [6]. This decline is attributed to a combination of peripheral and central factors. At the peripheral level, the regenerative capacity of olfactory receptor neurons diminishes with age, leading to a reduced sensory input [6]. At the central level, structural atrophy in olfactory-related brain regions has been documented in healthy aging populations [7]. However, this decline is not consistent across individuals. Inter-individual variability suggests that preserved olfactory function may serve as a marker of cognitive resilience. Indeed, older adults with intact olfactory abilities often exhibit superior cognitive outcomes and reduced risk of neurodegenerative diseases [8].

Research on olfaction has provided critical insights into the pathophysiology of neurodegenerative diseases, especially Alzheimer’s disease (AD) [5]. Indeed, the decline in olfactory abilities is more pronounced in individuals with dementia, particularly AD. Olfactory impairments, including deficits in odor identification, odor discrimination, and odor recall, often emerge in the preclinical stages of AD, preceding significant cognitive decline [9]. Furthermore, structural imaging studies reveal pronounced atrophy in the entorhinal cortex, amygdala, and hippocampus among individuals with olfactory dysfunction, aligning with known patterns of AD pathology [10,11,12]. Therefore, a growing body of research has focused on the role of olfactory dysfunction as a predictor of conversion from mild cognitive impairment (MCI) to AD.

MCI often represents a transitional phase between normal aging and dementia [13], with an annual progression rate to dementia ranging, in Italy, from 5% to 15%, depending on the setting and the diagnostic criteria applied [14,15]. However, predicting this conversion remains a challenge. Olfactory dysfunction has emerged as a robust predictor of this progression. Olfactory assessments consistently show greater impairments in odor identification, discrimination, and recall in individuals with MCI who later convert to AD compared to non-converters [9]. Structural imaging studies support these findings, showing atrophy in olfactory-related brain regions, particularly the entorhinal cortex and hippocampus [11,12]. Likewise, functional imaging studies show diminished activation and connectivity in olfactory and memory-related networks during olfactory tasks, including orbitofrontal cortex, anterior cingulate cortex, and the default mode network (DMN) in converters [16].

To the best of our knowledge, most of the previous studies investigated olfactory abilities solely through the Identification test from the Burghart’s Sniffin Sticks Test [9] or the University of Pennsylvania Smell Identification Test (UPSIT, MediSense). However, UPSIT includes odors that are typical of American culture, and its adaptations for other cultures are often unsuitable (e.g., the Italian version includes the scent of pasta, an odor that is absent in Italian culture). In addition, most of these cross-sectional studies compared different groups of patients, some with MCI and others with AD [17,18], limiting the ability to capture the dynamic trajectory of olfactory decline and its relationship with cognitive deterioration. This approach assumes homogeneity within MCI subgroups, failing to account for the substantial inter-individual variability in disease progression and olfactory impairment.

Here, we conducted a longitudinal study in a cohort of Italian MCI patients, assessing their olfactory capacities and brain morphology at the time of diagnosis, and at an average follow-up duration of 3.7 years (44.4 months), when some of the patients converted to AD. Our aim was to identify the patterns of cerebral atrophy and/or olfactory function that might predict the conversion from MCI to AD.

## 2. Materials and Methods

### 2.1. Participants

A total of 41 MCI patients (21 men; mean age 69.9 ± SD 7.9 years; education 10 ± SD 3.9 years) and 35 healthy controls (HC; 15 men; mean age 60.3 ± SD 7.5 years; education 13.3 ± SD 3.3 years; Table 1) took part in the study between February 2017 and July 2020. Data analyses were conducted on the 27 MCI patients (14 men; mean age 71.4 ± SD 7.5 years; education 10.2 ± SD 3.9 years) whose follow-up data were available (mean follow-up 44.4 months). Among these, thirteen MCI patients converted into Alzheimer’s disease patients (converter MCI, cMCI); the other fourteen patients were classified as stable MCI (sMCI). Clinical diagnoses of MCI and dementia due to AD were made according to published criteria [19,20], i.e., objective cognitive deficit in two tests of one cognitive domain or one test in two or more cognitive domains in the absence of impairment in daily activities as measured by Activities of Daily Living. Single- and multiple-domain MCI patients were recruited. The exclusion criteria were history of major functional psychiatric disorders, overt dementia, acquired and vascular brain injuries, ineligibility for MRI exam (cardiac pacemaker, nerve stimulators, cochlear implant, artificial crystalline lens, ferromagnetic prosthesis, mechanical stitches, and other metallic fragments, claustrophobia). In addition, while collecting the medical history, participants were asked about possible chronic rhinal disorders (nasal polyps, neuroepithelial tumors, septal reconstruction surgeries, chronic sinusitis) to exclude the presence of factors that could cause peripheral olfactory deficits. Smokers were excluded from the study.

Participants were all right-handed. At the time of the MCI diagnosis, the cMCI and sMCI subgroups were cognitively comparable (mini-mental state examination—mean MMSE scores: 26.5 vs. 27; not statistically different t(25) = −0.75, *p* = 0.5; Table 1).

The study was approved by the local ethics committee (Comitato Etico dell’Area Vasta Emilia Nord—107/2016/SPER/AOUMO, approved on 4 July 2016) and all participants provided written informed consents, in accordance with the Declaration of Helsinki [21].

### 2.2. Cognitive and Olfactory Assessment

Patients underwent both a standard clinical neurological assessment and a comprehensive neuropsychological evaluation (Table 2). The neuropsychological assessment was conducted in a single session lasting approximately 1 h and a half. All clinical diagnoses were made by specialists, including neuropsychologists and neurologists. On a subsequent day, the olfactory assessment was administered to all the participants (MCI and HC) by means of the Burgarth Sniffin’ Sticks Tests (MediSense; [22,23]) in a single session lasting between 30 and 50 min, depending on the individual participants’ abilities. This battery comprises three tests: Threshold (TT), Discrimination (DT) and Identification (IT) tests.

In the TT, participants are sequentially presented with triplets of the same odorant sticks in similar but not identical concentration. The olfactory threshold was defined as the mean of the last 4 turning points (that is, when the odorant concentration is changed), similarly to the staircase technique [24]. In the DT, subjects were required to compare triplets of different odors and to identify the one that smelled differently. The IT required subjects to label the 16 different familiar odors (orange, leather, cinnamon, mint, banana, lemon, licorice, resin, garlic, coffee, apple, clove, ananas, rose, anise, peach), choosing the right one within groups of four labels. Each test has a specific score, and the total score is the sum of all the scores. The olfactory battery also comprises normative data as a function of the age of the subjects [22,23].

**Table 2:** List of tests used in the neuropsychological evaluation.

COGNITIVE DOMAIN	TESTS
LANGUAGE	Boston Naming Test [25]
	Phonemics verbal fluency [26]
	Semantic verbal fluency [26]
CONSTRUCTIONAL PRAXIS	Rey–Osterrieth Complex Figure Test [27]
MEMORY	Babcock Story Recall Test [28]
	Paired-associate Words learning [28]
	Free and Cued Selective Reminding Test (FCRST), comprising Immediate Free Recall (IFR), Immediate Total Recall (ITR), Index of Sensitivity of Cueing (ISC) [29]
	Recall of Rey–Osterrieth Complex Figure Test [27]
	Digit Span test [30]
	Corsi’s Spatial Span test [30]
LEARNING	Corsi’s Spatial Supra-Span test [31]
REASONING	Raven’s Cognitive Progressive Matrices [32]
	Similarities Test (Wechsler Adult Intelligence Scale-IV) [33]
ATTENTION	Visual Search/Attentive Matrices test [34]
EXECUTIVE FUNCTION	Frontal Assessment Battery (FAB) [35]
	Cognitive estimates (time and weights) [36]
	Stroop test [37]
	Trial Making Test (TMT) [38]

### 2.3. Imaging Data Collection

High-resolution structural T1-weighted anatomical images were acquired for all participants on a Philips 3T Intera (TR = 9.9 ms; TE = 4.6 ms; in plane matrix = 256 × 256; voxel size = 1 × 1 × 1 mm^3^; 170 sagittal slices).

### 2.4. Data Analyses

#### 2.4.1. Behavioural Data Analyses

Statistical analyses were performed using TIBCO Statistica, Version 14.0.1, Jasp software, Version 0.17.1 and R Studio software, Version 4.2.2.

Analyses of data distribution using the Shapiro–Wilk test showed that Burghart Sniffin’ Stick Tests scores were not normally distributed.

Mann–Whitney non-parametric U tests were conducted on Burghart Sniffin Stick Tests scores to investigate differences between experimental groups. The statistical threshold adjusted for multiple comparisons (Bonferroni correction) was set at *p* = 0.002. A series of Spearman’s correlation tests were conducted between the Burghart Sniffin Stick Tests scores and the neuropsychological tests scores separately for each group. An analysis of covariance (ANCOVA) was conducted on Burghart’s Sniffin Stick Tests scores (TT, DT, IT) normalized in natural logarithm (ln) as within factors with age and education as covariates. Analyses were adjusted by Bonferroni corrections for multiple comparisons to account for the probability of committing type-1 errors.

Receiver Operating Characteristic (ROC) curve analyses were performed to evaluate the accuracy of the Burghart Sniffin Stick Tests and of the neuropsychological test scores in discriminating HC, sMCI, and cMCI in our experimental groups. DeLong’s test was used to compare the Areas Under Curve (AUCs) of the ROC curves, with the aim to assess whether one test was significantly more accurate than the other in discriminating between groups of patients.

#### 2.4.2. MRI Data Analysis

Data were analyzed with MatLab R2020a (MathWorks Inc., Natick, MA, USA) and SPM12 (Wellcome Trust Centre for Neuroimaging, http://www.fil.ion.ucl.ac.uk/spm/ (accessed on 15 January 2025).

Voxel-Based Morphometry (VBM; [39,40,41]) was conducted on structural MRI images as a whole brain technique for characterizing regional volume and tissue concentration differences between MCI patients and HC.

The following three preprocessing steps were applied:

1. tissue segmentation in gray matter—GM, white matter—WM, and cerebral spinal fluid—CSF;
2. spatial normalization, using the Dartel algorithm to build a group-specific template and to warp participants’ scans onto this template, creating the flow field, which stores the deformation information;
3. spatial smoothing with an isotropic Gaussian FWHM kernel of 8 × 8 × 8 mm.

The statistical analysis was implemented using an implicit mask with an absolute threshold value of 0.2, with the aim to successfully estimate the smoothness of the residuals, correct for the false-discovery rate, and mitigate the false positives. We used parametric tests for each voxel simultaneously to compare GM volumes in the three experimental groups using 2-sample t-test models. We accounted for age, education, and Total Intracranial Volume (TIV) by including these parameters in models.

Correlational analyses were also conducted at the whole-brain level adding the raw scores from the olfactory and the neuropsychological tests as covariates.

A double statistical threshold was applied to obtain a combined significance, corrected for multiple comparisons at α < 0.05 and *p* < 0.001, as computed by 3dClustSim AFNI routine, Version 24.2.06, using the “-acf” option (https://afni.nimh.nih.gov/pub/dist/doc/program_help/3dClustSim.html; accessed on 15 January 2025).

By means of Marsbar [42], volumes were extracted from a series of Regions Of Interest (ROIs, see in Section 3) derived from the volume comparisons at the whole-brain level in order to conduct ROIs and correlation analyses with scores from the olfactory and the neuropsychological tests.

## 3. Results

### 3.1. Socio-Demographic Results

MCI patients and HC differed from each other in age (t(60) = −5.8, *p* < 0.001) and in educational level (t(60) = −3.4, *p* = 0.002). On the other hand, considering the two subgroups of MCI patients, cMCI and sMCI did not differ from each other in age and education.

### 3.2. Behavioural Results

#### 3.2.1. Burghart Sniffin Sticks Tests

The scores of the MCI and HC groups differed significantly in DT (U = 185 *p* < 0.001; mean MCI score = 8.7 ± SD 2.5; mean HC score = 11.5 ± SD 2) and IT (U = 211.5 *p* < 0.001; mean MCI score = 9.1 ± SD 2.8; mean HC score = 11.7 ± SD 2.6). On the other hand, the TT scores did not differ significantly (*p* = 0.08). In addition, no significant differences were found between TT (*p* = 0.3), DT (*p* = 0.07) and IT (*p* = 0.2) tests between the two subgroups of patients, cMCI and sMCI. The ANCOVA (F_6,110_ = 1.9, *p* = 0.09) showed that the effects of education (*p* = 0.2) and age (*p* = 0.07) did not affect the between-group analysis.

#### 3.2.2. Receiver Operating Characteristic (ROC) Curve and Area Under Curve (AUC) Analyses

Analyses of ROC and AUC curves were used to evaluate the discriminatory efficacy to distinguish between the two patient groups. According to Swets’ classification, an AUC value of 0.5 indicates that the test is not informative, values between 0.5 and 0.7 indicate low accuracy, values between 0.7 and 0.9 suggest moderate accuracy, values from 0.9 to just below 1.0 signify high accuracy, and an AUC of 1 corresponds to a perfect test.

**Figure 1:**
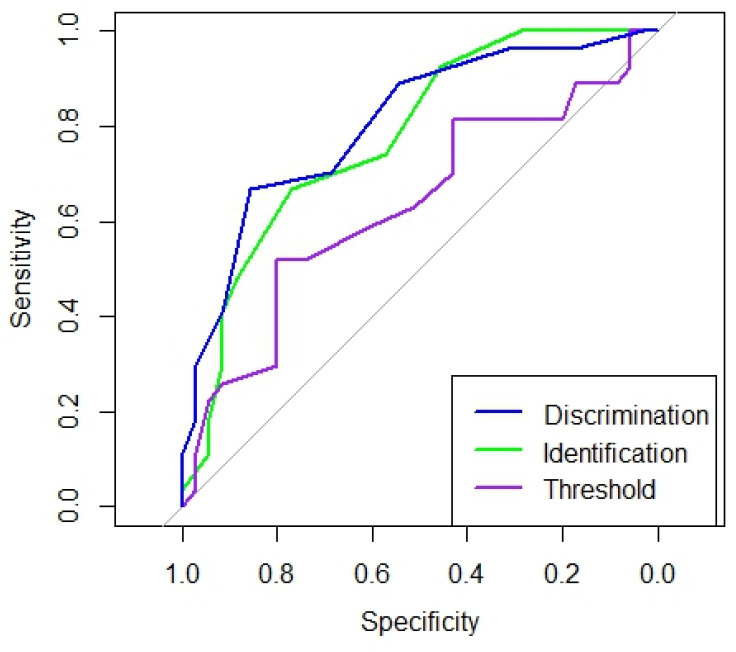
AUC values for the olfactory tests (Burghart Sniffin’ Sticks Tests).

The AUC value distinguishing HC from MCI patients for DT was 0.8 with a sensitivity of 66.7% and a specificity of 85.7%; for IT, it was 0.8 with a sensitivity of 66.7% and a specificity of 77.1%; and for TT, it was 0.6 with a sensitivity of 51.9% and a specificity of 80%. These results suggest that DT and IT are more accurate for the identification of MCI compared to TT (Figure 1).

**Figure 2:**
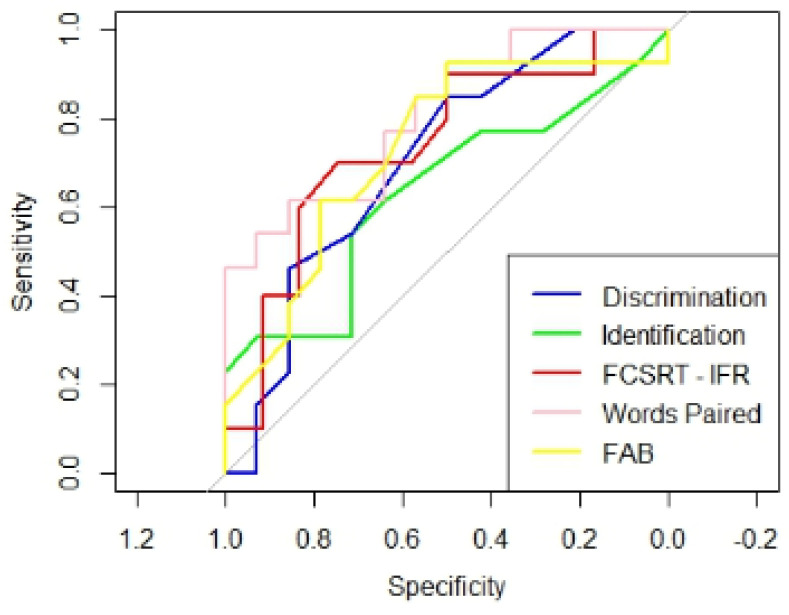
AUC values distinguishing cMCI from sMCI patients for the DT, IT, FCSRT—IFR, Paired-associate words and FAB scores.

On the other hand, the AUC value distinguishing cMCI and sMCI for DT was 0.7 with a sensitivity of 84.6% and a specificity of 50%; for IT, it was 0.6 with a sensitivity of 61.5% and a specificity of 64.3%; and for TT, it was 0.6 with a sensitivity of 46.2% and a specificity of 85.7% (Figure 2).

Regarding the neuropsychological tests, we considered the most sensitive tests for detecting the typical degeneration associated with AD, as reported in the literature. The AUC of the Immediate Free Recall (IFR) scores from the Free and Cued Selective Reminding Test (FCSRT), distinguishing cMCI from sMCI, was 0.7 with a sensitivity of 70% and a specificity of 75% (Figure 2); the Immediate Total Recall (ITR) and the Index of Sensitivity of Cueing (ISC) scores from the FCSRT both had an AUC of 0.6 with a sensitivity of 100% and a specificity of 42% (Figure 2). The AUC of the Paired-associate words test was 0.8 with a sensitivity of 62% and a specificity of 86% (Figure 2). Moreover, the AUC of the FAB was 0.7 with a sensitivity of 92,3% and a specificity of 50% (Figure 2).

The DeLong test showed that the comparisons between the AUCs discriminating cMCI and sMCI related to the DT and the AUCs related both to IFR scores (*p* = 0.9), Paired-associate words test (*p* = 0.6) and FAB (*p* = 0.8) were not significant. The comparisons between the AUC related to IT and the AUCs related to IFR (*p* = 0.2), Paired-associate words (*p* = 0.2) tests, and FAB (*p* = 0.5) were also not significant. Thus, in our data, the odor discrimination and identification tests (DT and IT), the IFR from the FCSRT, Paired-associate words test, and FAB were equally accurate in discriminating cMCI from sMCI. The TT did not prove particularly accurate in distinguishing between HC and MCI, nor between cMCI and sMCI in our cohort. Therefore, it was not compared with cognitive tests.

#### 3.2.3. Correlational Analyses Between Olfactory and Cognitive Assessment

The correlation analyses showed that in MCI patients, the DT and IT scores were significantly related to memory (Babcock Story Recall Test; Paired-associate words test; IFR, ITR and ISC from FCSRT; Recall of Rey–Osterrieth Complex Figure Test) and executive function (Cognitive Estimate) performance (Table S1 in Supplementary Materials).

Interestingly, when considering the two subgroups of MCI patients, we found strong correlations for both groups, but with different neuropsychological tests, which are reported in Table 3.

In summary, our results suggest that the odor performance, especially in the cognitive olfactory tests (DT and IT), at the early stage of dementia positively correlates with memory performance.

### 3.3. Voxel-Based Morphometry Results

**Figure 3:**
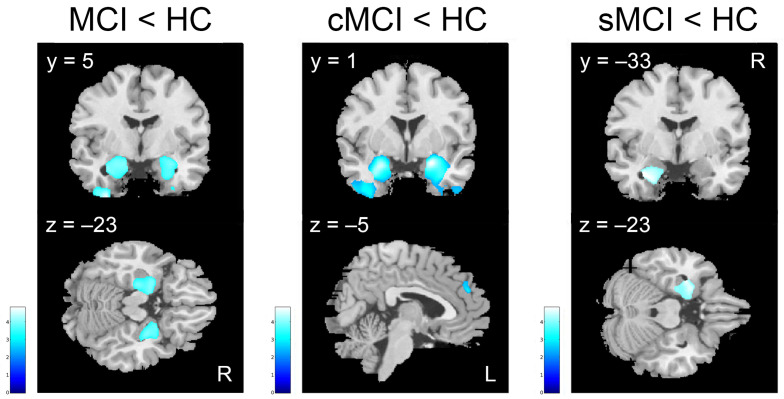
AUC values distinguishing cMCI from sMCI patients for DT, IT, FCSRT—IFR, Paired-associate words and FAB scores.

**Figure 4:**
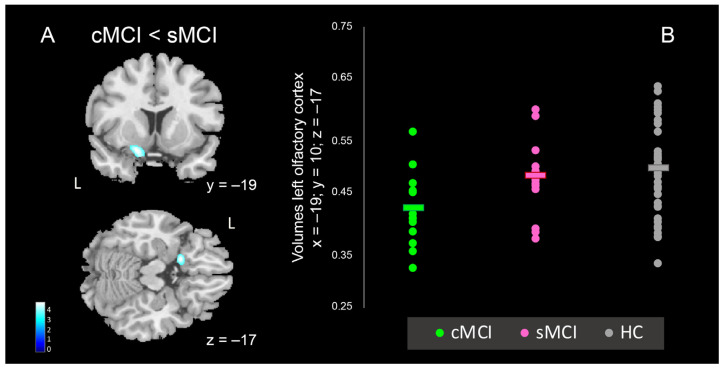
(**A**) Areas of significant volume reduction in cMCI patients compared to sMCI patients. Corrected for multiple comparisons at α < 0.05 (cluster size threshold k ≥ 652 voxels for *p* < 0.001). Blobs are superimposed on the SPM 12 template. (**B**) For illustrative purposes, box plots show volumes for left olfactory cortex ROI.

The VBM group analyses in MCI patients compared to HC showed significant areas of reduced gray matter (GM) volume bilaterally in the superior, middle, and inferior temporal gyri, comprising hippocampus and parahippocampal cortex, amygdala, fusiform, and lingual gyri (minimum cluster size k ≥ 543; Table 4, Figure 3). Similarly, the brain atrophy in sMCI patients compared to HC involved left medial temporal lobe and superior temporal gyrus (minimum cluster size k ≥ 538, Table 4, Figure 3). In cMCI patients in comparison to HC, the brain atrophy predominantly affected bilateral medial temporal lobe, comprising hippocampus, parahippocampal gyrus, and amygdala, as well as frontal areas such as inferior frontal gyrus and Insula and left olfactory cortex (minimum cluster size k ≥ 602, Table 4, Figure 3). More interestingly, when comparing the cMCI to the sMCI patients, a significant reduction in the GMV was found in the left inferior frontal gyrus, especially in the gyrus rectus and olfactory cortex (minimum cluster size k ≥ 652, Table 5 and Figure 4). When comparing sMCI to cMCI patients, no significant GMV reduction was found.

More interestingly, when comparing the cMCI to the sMCI patients, a significant reduction in the GMV was found in the left inferior frontal gyrus, especially in the gyrus rectus and olfactory cortex (minimum cluster size k ≥ 652, Table 5 and Figure 4). When comparing sMCI to cMCI patients, no significant GMV reduction was found.

### 3.4. ROIs and Correlational Analyses

The ROIs were selected using the peak coordinates of the clusters resulting from the contrasts of the group analyses (Table 6). The ROIs corresponded to the original clusters.

**Figure 5:**
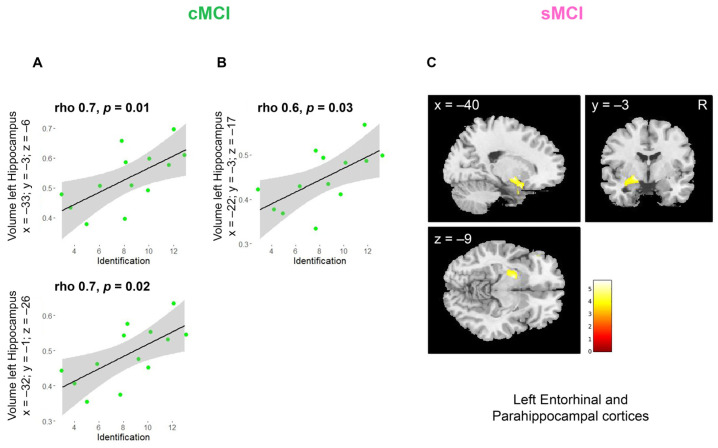
(**A**) Results of the correlational analyses between IT scores and volumes extracted from the ROI in the left hippocampus (HC vs. sMCI cluster peak x = −33; y = −3; z = −6; HC vs. MCI cluster peak x = −32; y = −1; z = −26) in cMCI. (**B**) Results of the correlational analyses between IT scores and volumes extracted from the ROI comprising both left hippocampus and olfactory cortex (HC vs. cMCI cluster peak x = −22; y = −3; z = −17). (**C**) Results of the whole-brain correlational analyses between IT scores and GMV in the left entorhinal and parahippocampal cortices (cluster peak x = −20; y = −3; z = −9, cluster size k ≥ 676) in sMCI. Blobs are superimposed on the SPM 12 template.

The correlational analyses conducted between the volumes extracted from the ROIs including left hippocampus (x = −33, y = −3, z = −6, rho = 0.7 *p* = 0.01; x = −32, y = −1, z = −26, rho = 0.7 *p* = 0.02) and the IT scores were positively correlated in cMCI patients (Figure 5A). Interestingly, in cMCI patients, the IT scores positively correlated also with volumes extracted from the ROIs including both left hippocampus and olfactory cortex (x = −22, y = −3, z = −17, rho = 0.6 *p* = 0.03; Figure 5B). Furthermore, in the whole-brain correlational analyses, we found that in sMCI the raw scores of IT positively correlated with the GMV of the left entorhinal and parahippocampal cortices (x = −20; y = −3; z = −9; cluster size 676, Figure 5C, Table 7).

**Figure 6:**
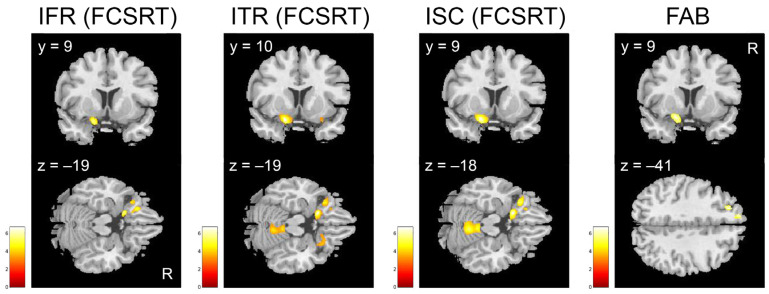
Results of the whole-brain correlational analyses between the indexes from FCSRT (IFR, ITR, ISC), the FAB scores and GMV in the left olfactory cortex in sMCI > cMCI. Cluster size threshold corrected for multiple comparisons at α < 0.05 and *p* < 0.001. Blobs are superimposed on the SPM 12 template. FCSRT = Free and Cued Selective Reminding Test, IFR = Immediate Free Recall, ITR = Immediate Total Recall, ISC = Index of Sensitivity of Cueing, FAB = Frontal Assessment Battery.

In addition, comparing sMCI to cMCI, we found that the IFR, ITR, and ISC indexes from FCSRT, as well as the scores from FAB positively correlated with the GMV of the left inferior frontal gyrus, especially comprising the olfactory cortex (Table 8; Figure 6).

## 4. Discussion

In the present study, we investigated the olfactory capacities of patients with Mild Cognitive Impairment (MCI) at the time of the diagnosis by means of the Burghart’s Sniffin Sticks Tests. Furthermore, we employed Voxel-Based Morphometry (VBM) to investigate the neural morphological correlates of olfactory capacities in these patients. Our aim was to identify the patterns of cerebral atrophy and olfactory function that might predict the conversion from MCI to Alzheimer’s disease (AD).

At the behavioural level, we found that MCI patients were impaired in the tasks requiring cognitive components, that is, in the discrimination (DT) and identification (IT) tests. The Receiver Operating Characteristics (ROC) and Areas Under Curve (AUCs) analyses for DT and IT demonstrated substantial accuracy in distinguishing MCI patients and HC. This finding is in line with previous studies which assessed olfactory dysfunctions in patients in the early stages of dementia ([5,18]; for a meta-analysis [9]). The group analysis did not reveal any significant difference between the two subgroups of patients. However, the more refined ROC and AUC analyses conducted on our patients’ cohorts revealed that DT and IT had remarkable accuracy in distinguishing between cMCI and sMCI. Notably, the discriminative capacity of these olfactory tests was comparable to those of the Paired-associate words test [28] and of the Frontal Assessment Battery (FAB; [35]), as well as that of the Immediate Free Recall (IFR) from the Free and Cued Selective Reminding Test (FCSRT, [43]). The FCSRT is widely recognized as a reliable tool for identifying individuals likely to convert to AD within five years following test administration, because of its strong predictive value [44,45]. As for DT and IT, they may help predict MCI evolution. Namely, these tests represent the cognitive components related to olfaction: discriminating odors (DT) requires episodic memory resources, since participants are exposed to a series of scents and later asked to determine which of these they encountered previously. Conversely, identifying odors (IT) relies on semantic memory, as it requires the participant to retrieve the correct verbal label for each scent [46]. In our cohorts, DT and IT scores are positively correlated with memory test scores only in sMCI patients, indicating that these olfactory tests may be useful for identifying individuals who will not convert to AD. Therefore, our results align with findings by Devanand and colleagues [47], who reported that odor testing enhanced the predictive value of global cognitive assessments and enabled the identification of individuals with a low likelihood of progressing to dementia. On the other hand, among converter MCI patients, the olfactory performance was linked to performance in executive function tests. Although executive functions were initially overlooked in AD research [48] because of the assumption that executive functions remained preserved in the preclinical stages of dementia [49,50], later studies demonstrated that these higher-order cognitive processes undergo early decline in the disease progression [51,52,53]. Namely, in MCI and AD patients, significant relationship was found between olfactory abilities and working memory, attentional shifting, attention, and decision making skills [54,55,56,57,58]. The relationship between neurodegeneration in AD and executive functions decline has been hypothesized to arise from their reliance on shared frontal brain regions, particularly the orbitofrontal cortex [59], susceptible to atrophy during the progression from MCI to AD [60].

The morphological deterioration found in our patients aligned with the typical trajectory of brain atrophy observed in MCI patients, with a predominant involvement of the medial temporal lobe degeneration. This pattern of atrophy has been widely recognized as a key factor in the origin and strongly correlated with the progression of dementia across all age groups [61]. Especially in AD, atrophy begins in the medial temporal lobe and progresses with defined stages [62]. Specifically, the entorhinal cortex is the first area to show atrophic changes, closely followed by the hippocampus, the amygdala, and the parahippocampus [63,64,65,66]. Our data are in line with these findings, since we found a similar volumetric reduction in stable MCI patients, whereas in converter MCI patients we found a larger atrophy, involving temporal and frontal areas and comprising bilateral olfactory cortex.

The volume of the olfactory bulb has been associated with odor identification [67] and odor detection thresholds [68], and the primary olfactory cortex volume with odor encoding of objects [69]. Previous studies showed that primary olfactory cortex and olfactory bulbs exhibit significant tau and amyloid deposition, which strongly correlates with deficits in olfactory functions [70], especially in AD patients [11]. Specifically, in a longitudinal study, Prestia and colleagues [71] found that patients who converted to AD showed greater atrophy in the primary olfactory cortex compared to participants whose cognitive impairment remained stable. Our results further corroborate the hypothesis of the predictive value of olfactory cortex degeneration in AD progression. Indeed, as shown by the ROC and AUC analyses, DT and IT were sufficiently accurate to differentiate cMCI from sMCI at the time of the MCI diagnosis, and converter MCI patients already exhibited significantly greater atrophy in the olfactory cortex compared to those who did not progress to AD.

Our data established a direct relationship between olfaction and memory. A positive correlation emerged between olfactory identification scores and left hippocampal, parahippocampal and entorhinal volume in both MCI patient subgroups. This is in line with several studies suggesting a functional interconnection between olfaction and memory and highlighting the critical role of their shared neural substrates (see [11] for a systematic review and [9] for a meta-analysis). Kamath et al. [8] examined olfactory identification deficits in 1600 older adults and found that these deficits were associated with grey matter volume of brain regions highly susceptible to neurodegeneration: in healthy controls, the amygdala’s volume emerged as the primary correlate of olfactory deficits, whereas in individuals with mild cognitive impairment, reduced odor identification abilities were linked to smaller volumes in the olfactory cortex, amygdala, entorhinal cortex, and hippocampus. Moreover, in sMCI, we found that a greater gray matter volume of the olfactory cortex correlates with better performance in verbal memory. On the other hand, we found that the gray matter volume of the olfactory cortex in sMCI and the olfactory behavioural performance in cMCI correlated with executive function. These results further suggest that compromised olfactory function may represent an early marker of underlying neurodegeneration and subsequent cognitive deterioration. The use of executive functions may be due to the involvement of the frontal areas as a compensatory mechanism for the olfactory cortex atrophy. This resembles the already known mechanism evidenced in healthy aging. Indeed, several studies have demonstrated that, with aging, older adults tend to recruit the frontal regions (and particularly prefrontal areas) more intensively to compensate for the diminished functionality of posterior brain areas. This phenomenon is well described by models such as the Posterior–Anterior Shift in Aging (PASA; [72]).

Summing up, our findings demonstrate that the combination of olfactory testing and gray matter volume assessment at the time of MCI diagnosis could effectively identify patients who will convert to AD.

### Limitations

While our study offers valuable insights into olfactory dysfunction and brain atrophy in the early stages of MCI, some limitations must be acknowledged. First, the sample size, though sufficient for the primary analyses, remains relatively small. Furthermore, we only tested an Italian cohort of MCI patients. These weaknesses could limit the generalizability of our current findings. Moreover, not all patients had available biomarker data, such as amyloid and tau imaging, which restricted our ability to fully investigate the possible relationship with molecular underpinnings of the observed olfactory deficits and brain atrophy. Additionally, although our study adopted a longitudinal design, due to the dropout of subjects, the follow-up period was relatively short.

## 5. Conclusions

In conclusion, our study significantly contributes to the growing body of literature on the role of olfactory dysfunction in the early stages of Mild Cognitive Impairment and Alzheimer’s disease. Our results demonstrate that deficits in olfactory functions, specifically odor discrimination and odor identification, in combination with structural data represent reliable biomarkers to differentiate between MCI patients and healthy controls. Furthermore, these tests may also help identify MCI patients who are less likely to convert to AD. Indeed, the atrophy affects the olfactory cortex in cMCI patients, but not in sMCI patients. On the other hand, in sMCI, the volume of this region correlates with memory capacity and executive functions. Therefore, these findings highlight the predictive role of the olfactory cortex in neurodegeneration and cognitive decline. Further investigations with larger, more diverse cohorts and longer follow-up periods are needed to track long-term changes in olfactory performance and brain morphology. Moreover, functional protocols including task-based fMRI using odor identification and recognition, as well as resting state functional connectivity, should be assessed as possible biomarkers of MCI to AD conversion.

To conclude, our results make a significant contribution to the field by suggesting that integrating non-invasive olfactory testing with structural MRI could lead to more accurate and timely predictions of Alzheimer’s disease progression, ultimately benefiting clinical practice and improving patient management.

## Figures and Tables

**Table 1:** Demographic and cognitive characteristics. Mean values and standard deviation values (in parenthesis) are reported. MMSE = Mini-Mental State Examination, cMCI = converter MCI, sMCI = stable MCI.

	Whole Group (n = 27)	cMCI (n = 13)	sMCI (n = 14)	Group Comparison
Age (years)	71.4 (±7.5)	71.9 (±7.4)	70.9 (±7.5)	*p* = 0.7
Gender F/M	14:13	7:6	7:7	*p* = 1
MMSE	27 (±2)	26.5 (±1.9)	27 (±1.8)	*p* = 0.5

**Table 3:** Results of the correlational analyses between olfactory and neuropsychological performance for the subgroups of patients. sMCI = stable MCI, cMCI = converter MCI, IT = identification test, DT = discrimination test, IFR = immediate free recall, ITR = immediate total recall, ISC = index of sensitivity of cueing, FAB = frontal assessment battery; * *p* < 0.05; ** *p* < 0.01; *** *p* < 0.001.

Group	Olfactory Battery Tests	Neuropsychological Tests	Spearman’s Rho
sMCI	DT	IFR	0.8 **
		ITR	0.8 **
		ISC	0.8 **
		Raven Matrices	0.6 *
sMCI	IT	IFR	0.9 ***
		ITR	0.9 ***
		ISC	0.8 ***
		Raven Matrices	0.8 **
		Boston Naming Test	0.7 **
		Stroop (Rate of Errors)	−0.6 *
cMCI	DT	Paired-Associate Words Test	0.6 *
		FAB	0.7 *
cMCI	IT	Weight Estimate Score	0.8 **
		Total Cognitive Estimates Scores	0.6 *

**Table 4:** Results of the VBM group analyses. Areas of significant volume reduction in MCI (cMCI and sMCI) patients compared to HC. Cluster size threshold corrected for multiple comparisons at α < 0.05 and *p* < 0.001. BA = Brodmann Area; L = left; R = right; MCI = Mild CognitiveImpairment; HC = healthy controls.

Anatomical Regions	BA	Side	Cluster	Voxel Level	MNI Coordinates		
**MCI < HC (k ≥ 543)**			**k**	**T**	**x**	**y**	**z**
Superior, Middle, Inferior Temporal gyri, Fusiform gyrus, Uncus	38, 21, 20	L	2907	4.81	−35	−3	−50
Hippocampus, Parahippocampal gyrus, Amygdala, Uncus, Superior Temporal gyrus, Fusiform gyrus, Temporal Pole	37, 38	L	6101	4.22	−32	−1	26
Hippocampus, Parahippocampal gyrus, Amygdala, Caudate, Uncus, Superior Temporal gyrus, Fusiform gyrus, Temporal Pole	37, 38	R	5832	4.17	28	5	−23
Superior, Middle, Inferior Temporal gyri, Fusiform gyrus, Uncus	38	R	1616	4.12	46	7	−47
**cMCI < HC (k ≥ 602)**			**k**	**T**	**x**	**y**	**z**
Hippocampus, Parahippocampal gyrus, Amygdala, Uncus, Precuneus, Superior, Middle, Inferior Temporal gyri, Temporal Pole, Fusiform gyrus, Lingual gyrus, Inferior Frontal gyrus, Olfactory cortex	20, 21, 27, 30, 47	R	17,225	6.99	22	1	−18
Hippocampus, Parahippocampal gyrus, Amygdala, Insula, Putamen, Uncus, Precuneus, Superior, Middle, Inferior Temporal gyri, Fusiform gyrus, Lingual gyrus, Temporal Pole, Inferior Frontal gyrus, Olfactory cortex, Cerebellum	20, 21, 27, 37, 38, 47	L	19,029	6.72	−22	−3	−17
Medial Frontal gyrus	6, 8, 9	L	656	4.22	−5	47	28
Cingulate gyrus	23, 24, 31	R	1225	4.18	1	−28	41
Insula, Putamen	44	R	712	4.16	35	12	3
Middle Temporal gyrus	21	L	768	4.08	−62	−28	−14
**sMCI < HC (k ≥ 538)**			**k**	**T**	**x**	**y**	**z**
Hippocampus, Parahippocampal gyrus, Amygdala, Uncus, Precuneus, Superior Temporal gyrus, Temporal Pole, Fusiform gyrus	38	L	2698	4.08	−33	−3	−26

**Table 5:** Results of the VBM group analysis in stable MCI (sMCI) vs. converter MCI (cMCI). Areas of significant volume reduction in cMCI patients compared to sMCI patients. Corrected for multiple comparisons at α < 0.05 (cluster size threshold k ≥ 652 voxels for *p* < 0.001). BA = Brodmann area; L = left; R = right.

Anatomical Regions	BA	Side	Cluster	Voxel Level	MNI Coordinates		
			k	T	x	y	z
Inferior Frontal gyrus, gyrus Rectus, Olfactory cortex, Insula	47	L	833	4.83	−19	10	−17
Middle, Inferior Temporal gyri, Middle Occipital gyrus	19, 37, 21	R	1002	4.8	54	−60	6

**Table 6:** Regions of interest (ROIs) selected from the main clusters of the VBM group analyses contrasts. VBM = voxel-based morphometry; HC = healthy control; sMCI = stable MCI; cMCI = converter MCI.

Anatomical Regions	Cluster Peak In MNI Coordinates			VBM Group Analyses
	**x**	**y**	**z**	**contrasts**
Left Hippocampus	−32	−1	−26	HC vs. MCI
Right Hippocampus	28	5	−23	HC vs. MCI
Left Hippocampus	−33	−3	−6	HC vs. sMCI
Left Hippocampus	−22	−3	−17	HC vs. cMCI
Right Hippocampus	22	1	−18	HC vs. cMCI
Right Insula	35	12	3	HC vs. cMCI
Left Medial Frontal Gyrus	−5	47	28	HC vs. cMCI
Cingulate Gyrus	1	−28	41	HC vs. cMCI
Left Olfactory Cortex	−19	10	−17	sMCI vs. cMCI
Superior Frontal Gyrus	−24	35	41	sMCI vs. cMCI

**Table 7:** Results of the correlational analyses between IT scores and the whole-brain GMV in sMCI. BA = Brodmann area; L = left. Cluster size threshold k ≥ 676 voxels, corrected for multiple comparisons at α < 0.05 and *p* < 0.001.

Anatomical Regions Per Test	BA	Side	Cluster	Voxel Level	MNI Coordinates		
			k	T	x	y	z
Parahippocampal gyrus, Amygdala, Hippocampus, Fusiform gyrus, Olfactory cortex, Inferior Frontal gyrus, Insula	28, 37, 20, 34, 47	L	4291	5.6	−20	−3	−9
Inferior and Middle Frontal gyrus	47, 45	L	688	4.4	−47	31	1

**Table 8:** Results of the correlational analyses between neuropsychological scores and whole-brain GMV in sMCI > cMCI. BA = Brodmann area; L = left; R = right; IFR = Immediate Free Recall; ITR = Immediate Total Recall; ISC = Index of Sensitivity of Cueing; FCSRT = Free and Cued Selective Reminding Test; FAB = Frontal Assessment Battery. Cluster size threshold corrected for multiple comparisons at α < 0.05 and *p* < 0.001.

Anatomical Regions Per Test	BA	Side	Cluster	Voxel Level	MNI Coordinates		
**IFR from FCSRT (k ≥ 681)**			**k**	**T**	**x**	**y**	**z**
Inferior and Middle Frontal gyrus, Olfactory cortex, gyrus Rectus, Insula	11, 47	L	2410	5.9	−19	9	−19
Superior, Middle and Inferior Temporal gyrus	19, 22, 37	R	961	5.8	52	−61	8
**ITR from FCSRT (k ≥ 691)**			**k**	**T**	**x**	**y**	**z**
Inferior and Middle Frontal gyrus, Medial Frontal gyrus, Olfactory cortex, gyrus Rectus, Insula	47, 11	L	4954	7.7	−19	10	−19
Superior Parietal Lobule, Precuneus	7	R	1320	5.8	20	−58	65
Cerebellum		R	2542	5.1	5	−54	−18
Superior, Middle and Inferior Temporal gyrus	39, 37, 22	R	1086	4.8	53	−60	7
Inferior Frontal gyrus, Olfactory cortex, gyrus Rectus, Insula, Superior Temporal gyrus, Amygdala	47, 25, 34,	R	831	4.3	19	20	−21
**ISC from FCSRT (k ≥ 701)**			**k**	**T**	**x**	**y**	**z**
Middle and Inferior Frontal gyrus, Olfactory cortex, gyrus Rectus, Insula	47, 11, 25	L	5104	6.8	−19	10	−18
Superior Parietal lobule, Precuneus	7	R	1312	5.8	20	−58	65
Cerebellum		R	3826	5.6	5	−53	−18
**FAB (k ≥ 685)**			**k**	**T**	**x**	**y**	**z**
Superior and Middle Frontal gyrus, Medial Frontal gyrus	9, 8	L	776	5.2	−24	35	41
Olfactory cortex, Inferior Frontal gyrus, gyrus Rectus, Insula, Medial Frontal gyrus	34, 47, 25	L	721	4.9	−19	9	−16

## Data Availability

The data presented in this study are available on request from the corresponding author. Data is unavailable publicly due to privacy and ethical restrictions.

## Supplementary Materials

The following supporting information can be downloaded at: https://www.mdpi.com/article/10.3390/brainsci15030310/s1, Table S1: Correlational analyses in MCI group.

## Abbreviations

The following abbreviations are used in this manuscript:

AD	Alzheimer’s disease
MCI	Mild Cognitive Impairment
cMCI	Converter Mild Cognitive Impairment
sMCI	Stable Mild Cognitive Impairment
HC	Healthy Controls
TT	Threshold Test
DT	Discrimination Test
IT	Identification Test
VBM	Voxel-Based Morphometry
DMN	Default Mode Network
UPSIT	University of Pennsylvania Smell Identification Test
SD	Standard Deviation
MMSE	Mini Mental State Examination
MRI	Magnetic Resonance Imaging
F	Female
M	Male
FCRST	Free and Cued Selective Reminding Test
IFR	Immediate Free Recall
ITR	Immediate Total Recall
ISC	Index of Sensitivity of Cueing
FAB	Frontal Assessment Battery
TMT	Trial Making Test
TR	Repetition time
TE	Echo time
ANCOVA	Analysis of Covariance
ln	natural logarithm
ROC	Receiver Operating Characteristic
AUC	Areas Under Curve
GM	Gray Matter
WM	White Matter
CSF	Cerebral Spinal Fluid
FWHM	Full Width Half Maximum
TIV	Total Intracranial Volume
ROI	Regions Of Interest
BA	Brodmann Area
L	Left
R	Right
MNI	Montreal Neurological Institute coordinate system
PASA	Posterior–Anterior Shifting in Aging
