# Supplementary material for: Olfactory Testing and Gray Matter Volume: A Combined Approach to Predict the Conversion to Alzheimer
Source: Brain Sci. 2025 Mar 15;15(3):310. doi: 10.3390/brainsci15030310 (PMC11940542; doi:10.3390/brainsci15030310)
Supplement: Supplementary file 1 [file brainsci-15-00310-s001.zip › brainsci-3510494-supplementary.pdf]

Supplementary Materials

Correlational analyses in MCI group

Table S1. Results of the correlational analyses between olfactory and neuropsychological performance, for the MCI group. IT= identification test, DT= discrimination test, IFR= immediate free recall, ITR= immediate total recall, ISC= index of sensitivity of cueing, FAB= frontal assessment battery; *p< 0.05; ** p< 0.01; *** p< 0.001.

Olfactory Battery tests	Neuropsychological tests	Spearman's rho
DT	Babock story recall test	0.5*
	Paired-associate words test	0.5**
	IFR	0.5*
	ITR	0.6**
	ISC	0.6**
IT	Paired-associate words test	0.5*
	IFR	0.6**
	ITR	0.5*
	ISC	0.5*
	Recall of Rey-Osterrieth complex figure test	0.6**
	Weight cognitive estimates	0.6**
	Total cognitive estimates	0.6**
